# Anti-fatigue effect of Amarkand on endurance exercise capacity in rats

**DOI:** 10.1186/s12906-016-0995-2

**Published:** 2016-01-22

**Authors:** Aarti N. Narkhede, Suresh D. Jagtap, Pallavi S. Nirmal, Shital A. Giramkar, Bhagyashri E. Nagarkar, Omkar P. Kulkarni, Abhay M. Harsulkar

**Affiliations:** Department of Herbal Medicine, Interactive Research School for Health Affairs (IRSHA), Bharati Vidyapeeth Deemed University, Pune-Satara Road, Pune, 411 043 Maharashtra India

**Keywords:** Amarkand, Anti-fatigue activity, Polyphenolic content, Validation

## Abstract

**Background:**

Amarkand tubers are routinely used by many Indian tribes as a specialized food for health and longevity but so far there is no scientific evidence for their activities. Taxonomically, Amarkand belong to genera *Eulophia* and *Dioscorea*.

**Methods:**

In this communication, comparative antifatigue potential of Amarkand was analyzed using forced swimming model in rats and evaluated using biomarkers of physical fatigue.

**Results:**

Methanol extracts of tubers of *D. bulbifera*, *E. ochreata*, *E. leghapanensis* and bulbils of *D. bulbifera* exhibited rich polyphenolic content. *D. bulbifera* bulbils and *E. ochreata* significantly prolonged the swimming endurance time. Creatine kinase and urea nitrogen were significantly reduced by treatment of *D. bulbifera* bulbils and *E. ochreata* as compared to negative control. *D. bulbifera* bulbils effectively increased creatine (*p* < 0.001), lactate dehydrogenase (*p* < 0.01) and hemoglobin (*p* < 0.001) compared to negative control. *D. bulbifera* bulbils and *E. ochreata* treatments significantly increased glycogen (*p* < 0.05, *p* < 0.01) and lowered malondialdehyde levels (*p* < 0.001) in muscles and in liver tissue compared to negative control.

**Conclusion:**

These results indicate that a treatment with *D. bulbifera* bulbils and tubers of *E. ochreata* facilitates aerobic glucose metabolism and endurance by improving various impairments associated with fatigue.

## Background

Fatigue is a complex metabolic phenomenon, which involves listless conditions. During fatigue, body faces difficulty in initiating or sustaining voluntary activities. It induces alteration in performance leading to decrease in muscular power and endurance, decreased motor skill performance and diminished physical as well as mental functions [1]. Accumulation of metabolic by-products like magnesium, inorganic phosphates, lactate, reactive oxygen species etc. can potentially contribute to fatigue during exercise and limit the performance. Physiological fatigue may also result due to metabolic disorders like diabetes, hyperthyroidism, anemia, high Body Mass Index, liver diseases etc. [2]. Regular exercise helps the body to function better, but strenuous or high intensity exercise may cause reactive oxygen species (ROS) production and exhaustion leading to oxidative stress, which often overwhelms body’s capacity to detoxify them. Fatigue is worthy of attention since it may cause various disorders related to bio-regulatory and immune system. Also, these disorders cause reduction in exercise intensity or even interruption of activity [3].

Tribal populations are rich in traditional knowledge about the plants available in their surrounding and their medicinal as well as food values [4]. There are certain groups of plants locally identified by a particular vernacular name but botanically they belong to different genera and family. Amarkand is one of such groups. Tubers of Amarkand are extensively utilized by many Indian tribes as a source of food and especially as medicine [5]. Ethanobotanical survey in Maharashtra conducted during 2004 to 2009 by Medicinal Plants Conservation Centre (MPCC) revealed that, there are multiple species recognized as Amarkand especially from genus *Eulophia* (Family Orchidaceae) and one from genus *Dioscorea* (Family Dioscoreaceae). However, with respect to taxonomical knowledge, there is still confusion in identification of species of *Eulophia* under the name Amarkand, which has rich food and medicinal value [6].

About 32 species of *Eulophia,* spread all over India, along with *Dioscorea bulbifera* are recognized as Amarkand by tribes, of which 21 species are medicinally important. *Eulophia* species are used as medicine fairly throughout the greater part of India; most of them are diverse but mainly tropical found up to 2200 m above sea level [7]. According to ayurvedic perspectives, these species have wide spectrum of uses like expectorant, anabolic, tonic, diuretic, astringent, digestive and soft purgative. It promotes strength and alleviates all the three ‘doshas’. It has also been used in ear discharge, blood clotting, joint edema and debility also [8]. According to ethno-botanical reports, tubers of these plants are described as appetizer, anthelmintic, aphrodisiac, stomachic, alterative and commonly given to stimulate appetite and to purify blood in heart troubles [9–11]. Pharmacologically, *E. ochreata* and *D. bulbifera* are reported for their antioxidant [12], anti-inflammatory [13], nutritional content [14], antihyperglycemic, antidyslipidemic [15], analgesic [16] activity.

In our earlier study, out of 21 species, 11 species of Amarkand were prioritized and studied for their free radical quenching potential, of which four species namely, bulbils of *D. bulbifera* and tubers of *E. ochreata, E. leghapanensis, D. bulbifera* have been prioritized and selected in this study [17]. Polyphenolic contents from plants are known to be helpful for extensive exercise [3], thus initially polyphenolic content of these selected species of Amarkand were analyzed. These three plants are still used by different tribes as they believe that these bulbils and tubers would maintain as well as increase their endurance for hard work and thereby extend their longevity [4, 17].

These species are used in routine diet and as a medicine by tribal people, but still it is less explored in urban lifestyle. Though the literature reveals, its high potential as a medicine as well as food, no study evidence has been reported on these species individually or comparatively concerning their anti-fatigue activity. Thus, in view to search complementary and/or alternative therapy from medicinal plants, it is obligatory to resolve the abstraction for Amarkand plants with reference to endurance or postpone the appearance of fatigue for its societal significance. In the present study, methanol extracts of Amarkand species were investigated for their anti-fatigue activity which may further provide scientific evidence for development of Amarkand species as a potential natural anti-fatigue agent. Considering the limitations of available therapies for fatigue in modern medicine [18], potential alternatives from traditional medicine and their mechanisms of action are worth investigating.

## Methods

### Plant material and extraction

Fresh tubers of *Eulophia ochreata* Lindl. (EO), *Eulophia leghapanensis* (Noven) (EL), *Dioscorea bulbifera* L. (DBT) and bulbils of *Dioscorea bulbifera* L. (DBB) were identified and collected by Dr. Suresh Jagtap, Botanist, from Leghapani (Nandurbar) and Bhimashankar (Pune) of Maharashtra, India. Plants were authenticated at Medicinal Plants Conservation Centre (MPCC), Pune with voucher specimen numbers, MPCC3125 for EO, MPCC3468 for EL and MPCC906 for *D. bulbifera*. The tubers were cut into small pieces and shade dried. The dried material was then pulverized using an electric blender and stored in an air tight polythene bags for further use. Methanol extract was prepared from these samples using hot extraction method followed by concentrating to dryness under reduced pressure using rotary evaporator.

### Determination of total phenolic, flavonoids and proanthocyanidin contents

Total phenol contents of all Amarkand tuber extracts were quantified using folin-ciocalteu reagent method mentioned in Singleton and Rossi, [19]. All four samples were analyzed at a final concentration of 50 μg/ml. Results are expressed as mg/g of gallic acid equivalent. Total flavonoid content was assessed according to [20]. Evaluation was carried out at the concentration of 1 mg/ml for all the samples. The flavonoid content is expressed as mg of quercetin equivalents/g of dry matter. Total proanthocynidin content was determined using procedure followed by [20]. All the extracts were evaluated at 1 mg/ml concentration and results are expressed as catechin equivalent (mg/g).

### Experimental animals

Approval for the experimental protocol from Institutional animal ethical committee was obtained before initiation of the study (CPCSEA/7/2679/2012-2013) from Bharati Vidyapeeth Deemed University, Medical College, Pune. Wistar rats (Female) weighing approximately 100–120 g were used for the study. They were housed for 2 weeks in solid bottomed polypropylene cages for acclimatization before use, maintained under standard conditions and fed standard rat chow with water *ad libitum*. Guidelines laid down by the CPCSEA were observed throughout the study for animal handling and experimentation. Animals were randomly divided into 14 groups having six rats per group. Low-100 mg/kg bw (L), Medium 300 mg/kg bw (M) and High 500 mg/kg bw (H) dose of plant extracts including sedentary and control group. Sedentary and control group were fed with distilled water by gavage and other 12 treated groups were force fed with different doses of EO, EL, DBT and DBB (100, 300 and 500 mg/kg bw) by gavage for 2 weeks.

### Weight loaded forced swimming test

Weight loaded forced swimming test (FST) was performed according to Venuprasad et al., [21] with some modification. Amarkand treated and control rats were subjected to FST every alternate day supporting constant load with a lead block weighing approximately 10 % of their body weight attached to their tails. The attached weight forced these animals to maintain rapid continuous leg movements [22]. Exhaustion was determined by observing their loss of coordinated movements and failure to swim. Endurance time was recorded until the rat was completely exhausted and failed to return to surface to breathe within 10s [23]. FST was performed in small tank of 30 cm deep, containing water at temperature 25 ± 2 °C.

### Biochemical assays

After a period of 4 weeks, animals were sacrificed. Blood samples were collected from heart immediately after last exercise. It was collected by heparinized syringe into vacutainers. Whole blood was used to analyze hemoglobin, which was expressed in gm% and serum was separated by centrifugation at 3000 rpm at 4 °C for 10 min. Serum was used to analyze Serum Urea Nitrogen (SUN), Lactate Dehydrogenase (LDH), Creatine Kinase (CK) and Creatine (Cr). All these parameters were assessed according to the procedure provided by commercially available kits manufactured by Span diagnostic Ltd., India. SUN and Cr were expressed in mg/dl while LDH and CK were expressed in lu/L. Liver and muscle tissue from hind limb were collected and frozen immediately at −80 °C for estimation of MDA and glycogen content. MDA content was performed according to the method described by Vinodini et al., [24] and it is expressed in nm/g of tissue, whereas glycogen level in muscle/liver were analyzed using a kit (Cayman chemical company, USA) and it indicated as mg/g of tissue.

### Statistical analysis

The values are presented as mean ± SD. One way ANOVA was used to determine significant differences in groups and negative control group. All analyses were performed using Graph pad Prism 5 software and *p* values <0.05 were accepted as statistically significant.

## Result and discussion

Fatigue is a complex metabolic state. It has been demonstrated that strenuous exercise leads to increased production of reactive oxygen species which is assistive in dilating blood vessels so as to increase blood flow and supply of oxygen and glucose. However, prolonged state of muscular activity may lower the cellular function by affecting its integrity thereby considered responsible for muscular fatigue during exercise [25]. It is therefore therapeutically attractive to increase intake of antioxidants, which may reduce fatigue and improve endurance as well as performance during exhaustive exercise [26]. Polyphenolic content of selected extracts of Amarkand species are given in Table 1. EO extract showed highest flavonoid and proanthocnidin content, which is accordance with the earlier study that includes high polyphenolic content of EO [12]. While, DBT showed presence of high phenolic content. Overall this data indicates that all the studied plants are rich source of polyphenols.

**Table 1:** Polyphenolic content of methanolic extract of selected Amarkand plants

No.	Plant Extract	Polyphenolic content assays		
		Phenol content	Flavonoid content	Proanthocynidin content
		mg/g dry mass		
1	DBB	1.29 ± 0.03	1.43 ± 0.06	3.53 ± 0.15
2	DBT	1.45 ± 0.23	0.39 ± 0.06	3.80 ± 0.10
3	EO	1.72 ± 0.03	0.82 ± 0.09	9.24 ± 0.50
4	EL	0.61 ± 0.07	0.18 ± 0.02	0.23 ± 0.03

Values are mean ± SD (*n* = 3) and all results are equivalent to respective standards

*DBB - Dioscorea bulbifera* bulbils, *DBT - Dioscorea bulbifera* tubers, *EO - Eulophia ochreata*, *EL - Eulophia leghapanensis*

**Fig. 1:**
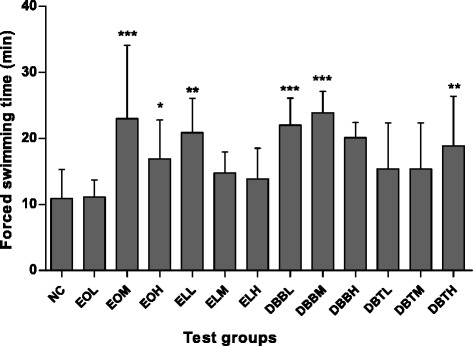
Effect of extracts of Amarkand tubers on forced swimming capacity in rats on the day 14. Values are expressed as Mean ± SD; *n* = 8. * *p* < 0.05; ** *p* < 0.01; *** *p* < 0.001 and compared to NC

FST is the most common method frequently used for the evaluation of anti-fatigue properties of test compounds. It can describe the gradual decrease in the force capacity of muscle or the endpoint of a sustained activity, and it can be measured as a reduction in muscular force or exhaustion due to contractile function [1]. To standardize the workload and reduce the swimming time, weights at specific body weight percentages were added to the tail of the animal [27]. Lower and medium doses of DBB extract and EOM treatment groups showed significant increase in swimming time to exhaustion as compared to negative control group was evident after 2 weeks (Fig. 1). Data presented herein indicate that DBB prolongs the swimming time of rats and improved endurance exercise capacity, this activity can be attributed flavonoid and proanthocynidin content in them.

Serum urea nitrogen is the product of energy metabolism. Protein and amino acids undergo stronger catabolic metabolism when the body cannot drive energy from sugar and fats [28]. A delicate balance exists between digestion of proteins and removal of urea from the body. It was reported that SUN is a sensitive parameter to evaluate the competence of human to tolerate physical load. Thus reduced SUN reflects reduced protein metabolism followed by enhanced endurance [29]. Thus, there is direct correlation between serum urea nitrogen in vivo and exercise tolerance. As shown in Table 2, after swimming, content of SUN in EO treated groups were significantly lower (19.46 ± 0.884 mg/dl), followed by DBB treated group as compared to negative control group (26.43 ± 4.928) and other Amarkand extracts treated groups. These results suggest that EO treatment reduces decaying of nitrogenous substances in the body and increased capacity to recover from fatigue.

**Table 2:** Effect of Amarkand plants on Creatine content, Hemoglobin concentration and Serum urea nitrogen in rats

Sr No	Treatment	Creatine (Cr)	Haemoglobin (Hb)	Serum Urea Nitrogen (SUN)
		mg/dl	gm%	mg/dl
1	HC	1.01 ± 0.122	13.16 ± 0.917	23.58 ± 2.916
2	NC	0.95 ± 0.052	12.28 ± 0.966	26.43 ± 4.928
3	EOL	1.05 ± 0.074	13.62 ± 1.137	21.20 ± 1.513*
4	EOM	1.21 ± 0.045***	14.13 ± 0.771**	19.46 ± 0.884***
5	EOH	0.81 ± 0.085	13.72 ± 0.608*	20.66 ± 1.676**
6	ELL	1.06 ± 0.080	13.28 ± 0.722	27.53 ± 3.794
7	ELM	1.09 ± 0.111	13.10 ± 1.203	21.20 ± 1.513*
8	ELH	1.14 ± 0.083*	13.37 ± 0.504	30.80 ± 2.343
9	DBBL	1.04 ± 0.175	13.75 ± 0.187*	26.20 ± 1.600
10	DBBM	1.27 ± 0.088***	13.35 ± 0.736	22.40 ± 4.498
11	DBBH	1.22 ± 0.172***	13.30 ± 0.328	19.71 ± 2.761**
12	DBTL	1.01 ± 0.080	13.18 ± 0.865	25.83 ± 2.163
13	DBTM	0.96 ± 0.075	13.32 ± 0.708	23.13 ± 1.420
14	DBTH	1.14 ± 0.027**	13.27 ± 0.671	28.98 ± 3.950

In front of all plant codes, abbreviations of their doses are given as, L - lower dose, M - middle dose, H - higher dose

All values are Mean ± SD where *n* = 6, * *p* < 0.05; ** *p* < 0.01; *** *p* < 0.001 compared to NC

Creatine in the form of phosphocreatine provides a quick energy source for the cells during exercise. Creatine is often taken as an energy-enhancing, fatigue-reducing supplement [30]. In the Table 2, DBB and EO at 300 mg/kg treated groups have shown significant increase in creatine content as compared to negative control group (*p* < 0.001), which indicate that EO and DBB treatments may boost energy level and increase resistance to muscular fatigue. Similarly, hemoglobin concentration below normal levels results in impaired oxygen delivery to the tissues and negatively affects endurance exercise capacity [22]. EO and DBB treated groups have shown significant increase in hemoglobin concentration, which is 14.13 ± 0.771 gm% (*p* < 0.01), 13.75 ± 0.187 gm% (*p* < 0.05), respectively as compared to negative control group, improving their efficacy to recover from fatigue.

**Fig. 2:**
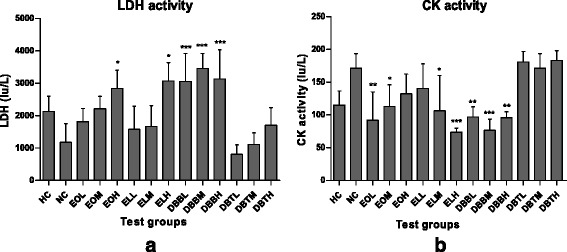
Effect of Amarkand plants on **a**) LDH activity and on **b**) CK activity in rats. Values are expressed as Mean ± SD; *n* = 8. * *p* < 0.05; ** *p* < 0.01; *** *p* < 0.001 compared to NC

Lactate dehydrogenase (LDH) and Creatine Kinase (CK) levels in serum are key indicators of fatigue. Increased levels of LDH and decrease in CK considered as an indication of cellular necrosis and tissue damage [31]. Conversion of pyruvate to lactate is the main energy source required for intense exercise in a short time under anaerobic condition [2]. Lactate drops the intracellular pH by accumulating within the myocyte, excess lactic acid however, inhibits the contractile activity as well as glycolysis in muscle tissue [32]. Therefore, LDH level is an important marker for the degree of fatigue and workload intensity. After swimming, lactate dehydrogenase levels in DBB treated groups for all the three doses were significantly higher (*p* < 0.001) than negative control groups and compared to other studied treated groups (Fig. 2).

On the contrary, exercise bouts leads to increase serum CK levels which gradually go back to basal level. Serum CK level is a marker of the functional status of muscle tissue, which also indicate cellular necrosis and acute or chronic muscular injuries. Significant decrease (*p* < 0.001) in CK level was also observed in EL and DBB treated groups in comparison with negative control group (Fig. 2). Venuprasad et al. [21] indicated lowering of CK levels in *O. sanctum* extract treated rats. This was attributed with the antifatigue activity of these extracts. These results indicate the beneficial effect of EL in fatigue by accelerated clearing lactate from cells as well as efficient energy utilization with potential scavenging of free radicals.

**Fig. 3:**
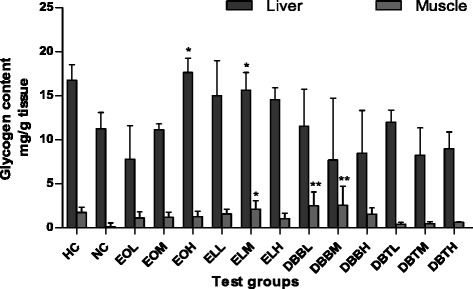
Effect of extracts of Amarkand tubers on glycogen content in liver and muscle tissues of rats. Values are expressed as Mean ± SD; *n* = 8. * *p* < 0.05; ** *p* < 0.01 compared to NC

Glycogen content is an integral determining factor in fatigue. Glycogen is a polysaccharide structure, which represents main storage form of glucose; it functions as the secondary long-term energy storage and forms an energy reserve that can be quickly mobilized to meet a sudden need of glucose [32]. Many studies have observed significant depletion of glycogen in both liver and muscles during exhaustive exercise, which is responsible for the fatigue [3]. After swimming, the concentration of liver glycogen of EO and EL treatment groups were significantly higher than that of negative control group (*p* <0.05). Carbohydrate-protein supplement is most beneficial during short recovery periods by enhancing the storage of muscle glycogen [33], and it was reported that EO tubers are promoted as a carbohydrate supplement [14]; it is a source of fibres, proteins and contribute greatly towards meeting human nutritional requirements [34]. Muscle glycogen is the major fuel used during sustained exercise. Depletion of glycogen results in reduction in the rate of ATP regeneration and impaired contractile activity of the muscle [35]. DBB (*p* <0.01) followed by EL (*p* <0.05) treatment groups showed significant increase in muscle glycogen than that of the negative control group (Fig. 3). As previous studies showed that *D. bulbifera* have antihyperglycemic, antidyslipidemic effect by lowering glycemic index, it provides more sustained form of energy and better protection from obesity and diabetes [15]. Our data indicates that administrations of EO, EL and DBB extracts significantly increases glycogen reserves in liver as well as muscle or at least minimize its utilization.

**Fig. 4:**
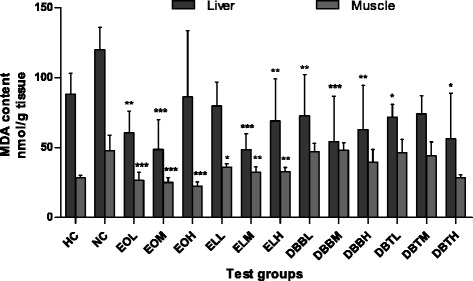
Effect of extracts of Amarkand tubers on MDA content in liver and muscle tissues of rats. Values are expressed as Mean ± SD; *n* = 8. * *p* < 0.05; ** *p* < 0.01; *** *p* < 0.001 compared to NC

Lipid peroxidation is an indicator of oxidative damage. It has been implicated in a variety of diseased state and responsible for increasing inflammation [36]. Metabolite produced during the lipid peroxidation process can be used as the method to attribute oxidized injury. Malondialdehyde (MDA) is a product of both lipid peroxidation and of prostaglandin biosynthesis. Thus its evaluation indicates oxidative damage to cell membrane lipids [37]. The content of MDA in liver tissue for DBBM and EOM treated groups were significantly lower than that of the control group (*p* <0.001). Similarly, EO treatment effectively lowers the MDA content in muscle tissues (*p* <0.001) Fig. 4. This study has revealed a significant effect on lipid peroxidation by EO and DBB treatment in rats which suggests its protective effect. It was described earlier by Pessoa et al., [38], that as a natural antioxidant, some components of DBB upregulate antioxidant enzyme activities by reducing lipid peroxidation as DBB also have analgesic and anti inflammatory potential [16].

In summary, our data suggested that methanol extracts of DBB, EO and EL has highest polyphenolic content. Presence of polyphenols may suggest their involvement in the anti-fatigue effects in rats. Further, DBB, EL and EO treatment extend the swimming time to exhaustion by activating the energy metabolism by increasing or maintaining the content of glycogen in liver and muscle. They help eliminate the accumulated products like SUN, lactic acid and decrease the CK activity as well as inhibit lipid peroxidation in liver and muscle tissues. They also maintain the Cr and Hb content, which may facilitate recovery from fatigue.

## Conclusions

Our data validated unexplored Amarkand group of plants, which are rich in components having various health benefits. Among four selected Amarkand species, DBB and EO supplementation may decrease the contribution of exercise-induced oxidative stress, and effectiveness to combat fatigue. Further studies are needed at the cellular and molecular levels to know their mechanism by means of understanding their role in regulation of glucose transport genes or through inflammatory pathways against fatigue and exercise endurance. Such type of study is required for societal benefit and sustaining the knowledge of tribal communities.
